# Lithic Miniaturization Provides a Signature of an MIS4‐3 Southern Dispersal of *Homo sapiens*

**DOI:** 10.1002/evan.70027

**Published:** 2026-03-06

**Authors:** Ceri Shipton

**Affiliations:** ^1^ School of Culture, History and Language, College of Asia and the Pacific The Australian National University Canberra Australian Capital Territory Australia; ^2^ Institute of Archaeology University College London London UK

**Keywords:** Asitau Kuru, bipolar knapping, Laili, laminar blades, microliths, Panga ya Saidi, Shi'bat Dhiya, Upper Paleolithic

## Abstract

Fossil and artefactual evidence shows *Homo sapiens* in Eurasia well before 75 ka. However, genetic evidence suggests all extant non‐African populations derive almost all of their ancestry from a dispersal that only diverged in the last 60–50 ka. In northern Eurasia, the Upper Paleolithic with its laminar blade knapping provides an archeological signature of this dispersal, but no equivalent is yet established for southern Asia, Wallacea, and Sahul. This paper suggests that lithic miniaturization may provide such a signature as it appears across these southern regions from around 50 ka. It can be traced back to the southwestern edge of Asia at 55 ka, and then coastal east Africa at 68 ka. In both these cases it is also associated with laminar blade technology. Lithic miniaturization is implicated in behaviors including bow‐and‐arrow hunting, compound tools, hair‐shaving, and scarification. The ecological and social implications of these behaviors may have given later *Homo sapiens* a competitive advantage over both other hominins and earlier dispersals of our own species.

## 1 Introduction

Our species dispersed out of Africa on multiple occasions over the course of its ~300 ka history (e.g., [1, 2, 3]). However, genetic evidence points to the primacy of a dispersal within the last 75 ka in giving rise to the great majority of extant non‐African human ancestry [4, 5, 6, 7]. The Upper Paleolithic with its characteristic laminar blade lithic technology has long been viewed as the archeological signature of this dispersal in western and northern Eurasia [8, 9]. But finding an equivalent signature in south‐eastern Eurasia, Wallacea, and Sahul, as well as a source in Africa, has proved problematic [10, 11, 12, 13]. This review proposes a new hypothesis for a universal signature of this dispersal from Africa across southern Eurasia and into Sahul: lithic miniaturization. Such a signature may even be partly reflected in some Upper Paleolithic cultures. An explanation is offered as to why a dispersal characterized by lithic miniaturization replaced both other hominin species and previous dispersals of *Homo sapiens*.

### 1.1 Defining Miniaturization

Lithic miniaturization is the deliberate creation of small stone flakes of fine‐grained (cryptocrystalline) material [14, 15, 16]. Deterministic perspectives on lithic technology, viewing size and material as dependent on available clasts, have led to these properties being overlooked as a culturally significant. However, recent studies have shown that material selection and lithic size are not solely determined by availability: Knappers almost always have a range of materials to choose from in their procurement territories and small flakes are sometimes produced even when large clasts are easily available [17, 18]. Moreover, when viewing lithics from a functional perspective, size and material are highly salient properties (e.g., [19, 20]).

**FIGURE 1:**
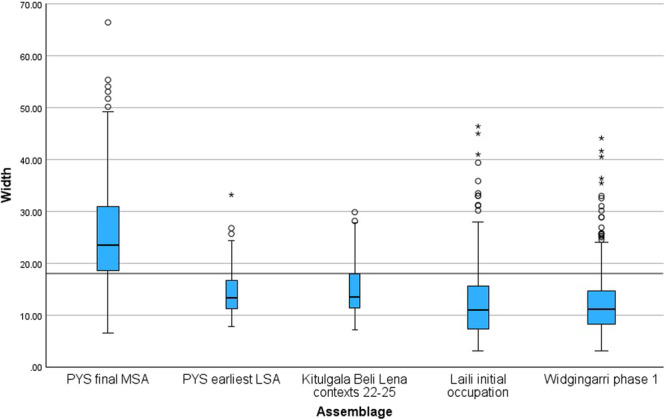
Boxplot of complete flake medial width in mm for the final MSA (layer 17) and the earliest LSA (layer 16) at Panga ya Saidi in east Africa [17]; alongside the early occupation at Kitulgala Beli Lena (contexts 22–25), Sri Lanka [23]; the initial occupation layer (20) of Laili, Timor‐Leste [18]; and the early occupation (phase 1) of Widgingarri 1, Australia [24]. Reference line is at 18 mm. Box widths are scaled to assemblage size. An ANOVA showed the between assemblage variation to be significant at *p* < 0.001 (df = 738, F = 83.489), with post‐hoc LSD tests showing the PYS final MSA to be significantly different to all other assemblages at the *p* < 0.001 level.

A definition of what constitutes small is complicated by taphonomic processes such as winnowing, as well as recovery methods, in particular sieve mesh size. Fine meshes have the potential to capture numerous flakes < 5 mm in length [21], indicating that coarser meshes may be distorting miniaturized assemblages. A lack of standardization in reporting of flake sizes necessitates a variety of measures of miniaturization. Length is the most reported aspect of lithic size, so a mean complete flake length of < 20 mm serves as a working threshold, however this is often the cut‐off used for sampling in lithic analysis, so again miniaturization may be widely underestimated. A length‐based threshold also has the disadvantage of being biased against blade focussed assemblages, so a mean complete flake weight of < 3 g could serve as an alternative definition [22], or a mean medial width of < 18 mm (Figure 1). Due to the influences of taphonomy, recovery methods, and reporting, miniaturization is most clearly demonstrated where it occurs in the same sequence or region as non‐miniaturized assemblages, and therefore is more likely to have been a hominin choice rather than a necessity. While there are multiple instances of the production and use of small lithics in the Lower and Middle Paleolithic (see Box 1), these seem to be explained by the restrictions of available clast sizes, and assemblages that meet the above size thresholds are scarce or absent [15].

BOX 1 **Early examples of small stone tools.**

Small tools were certainly used by Lower Paleolithic hominins, with several examples of butchery using small flakes [25, 26]. In the Levant, both the Oldowan site of Bizat Ruhama and the late Acheulean site of Revadim include a core‐on‐flake knapping strategy systematically producing small flakes, perhaps due to small chert clast size [27, 28]. Likewise, bipolar reduction—flaking through cracking stones between a hammer and anvil—of flint pebbles was used to produce small flakes at the Acheulean site of Thomas 1 Quarry in Morocco [29]. However, the average flake length in all these assemblages is in the 20–30 mm range rather than below 20 mm.

In the Middle Paleolithic, systematic small flake production and use has been documented at numerous sites such as Tata in Hungary [30] and Quebrada in Iberia [31], but in these cases mean flake length exceeds 20 mm. Bolomor Cave in Iberia presents an unusual case of numerous retouched small tools produced from pebbles, but there is not a focus on cryptocrystalline materials [32].

**FIGURE 2:**
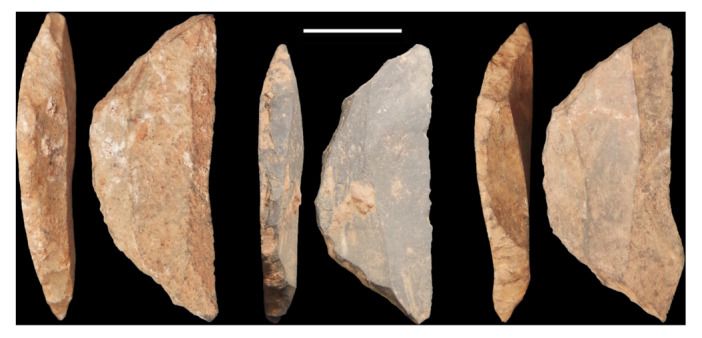
Backed crescents from the early MIS3 occupation (layers 11 and 12) at Panga ya Saidi. Note the small retouch on scars on the curved side of the artifacts creating a blunt edge. Scale is 1 cm.

The most common retouched tool types associated with miniaturized assemblages are backed microliths (Figure 2)—tools with one edge steepened for hafting with mastic, often used in multiples in a compound arrangement as in barbs on a harpoon or sickle blades. A previous hypothesis proposed that such tools were a signature of a *Homo sapiens* southern dispersal based on their presence in southern Africa and the Indian Sub‐continent in MIS4 and MIS3 respectively [33]. However, these are a repeatedly reinvented tool type [34] and likely convergent in these regions, with their presence in‐between being discontinuous [35]. They are not found east of India in MIS3 and so cannot provide a universal signature of a southern dispersal [34].

## 2 Africa

### 2.1 Panga ya Saidi

**FIGURE 3:**
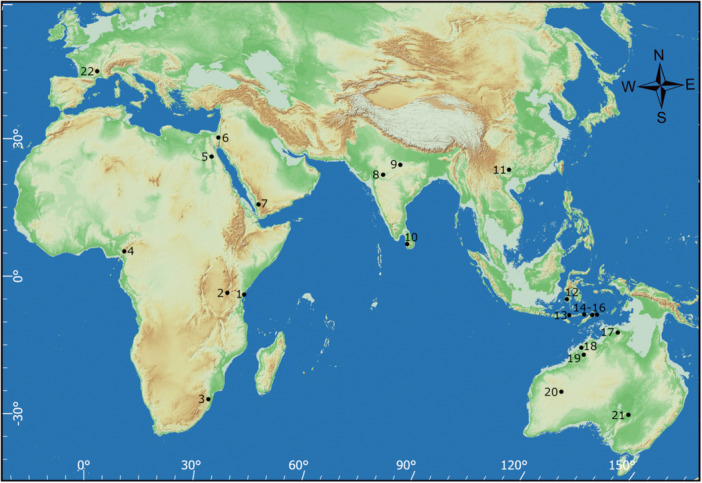
MIS3 sites with confirmed or probable miniaturized lithics shown on a −60 m sea‐level basemap to represent the early MIS3 coastline. GEBCO (General Bathymetric Chart of the Oceans) 2019 data, −60m contour. Terrain‐Elevation base layer provided by ArcGIS Online. 1. Panga ya Saidi; 2. Mumba; 3. Border Cave; 4. Shum Laka; 5. Taramsa Hill; 6. Boker Tachtit; 7. Shi'bat Dhiya; 8. Mehtakheri; 9. Dhaba; 10. Fa Hien Lena & Kitugala Lena; 11. Yahuai Cave; 12. Leang Bullu Bette & Leang Burung 2; 13. Liang Bua; 14. Makpan; 15. Laili; 16. Asitau Kuru; 17. Madjedbebe; 18. Widgingarri; 19. Carpenters Gap 1; 20. Karnatukul; 21. Warratyi; 22. Grotte Mandrin.

**FIGURE 4:**
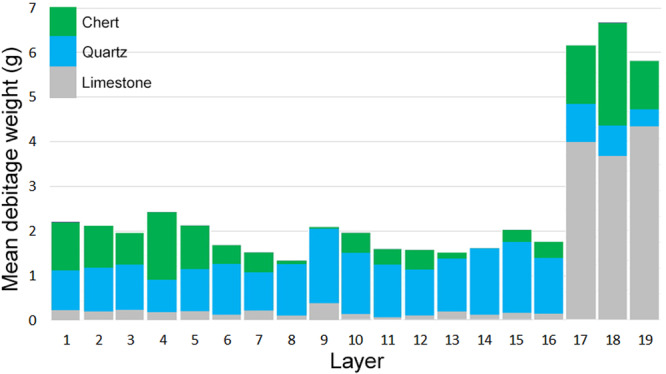
Mean debitage weight and material by layer at Panga ya Saidi.

In east Africa, the most complete Late Pleistocene archeological sequence is found on the Kenyan coast at the cave of Panga ya Saidi (Figure 3), which contains evidence of occupation in each of the last five marine isotope stages (MIS) [22]. At the end of MIS5 there is a classic Middle Stone Age (MSA) assemblage featuring Levallois technology, in association with a *Homo sapiens* burial [17, 36]. The molar morphology of this fossil is more similar to recent *Homo sapiens* than the late MIS5 representatives of our species outside of Africa from Qafzeh cave. This occupation at Panga ya Saidi is followed by a thick layer of very sparse MSA occupation at the MIS5‐4 transition. Then, in MIS4 (~68 ka), there is an abrupt shift from moderate‐sized coarser‐grained limestone as the dominant lithics, to small cryptocrystalline quartz and chert flakes, that is, miniaturization (Figures 1 and 4). The reduction in size is independent of the change in materials as it occurs even within quartz and chert artifacts [17]. This switch to miniaturization is unidirectional, characterizing the 15 subsequent occupation layers at Panga ya Saidi across MIS4‐1 (Figure 4). Miniaturization is also independent of technology, with it persisting across layers that are variously dominated by bipolar, Levallois, and laminar flaking.

**FIGURE 5:**
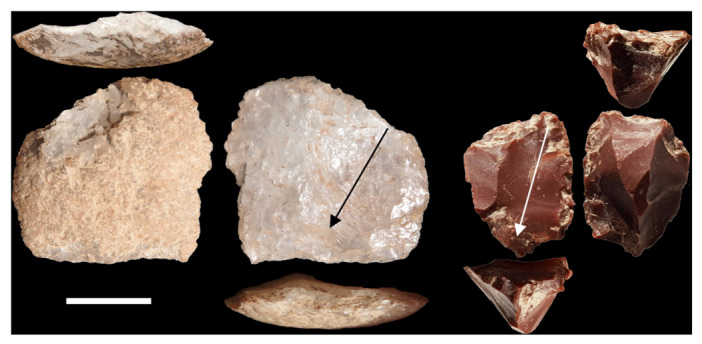
Quartz bipolar core from mid MIS3 at Panga ya Saidi (left); and chert bipolar core from MIS3 at Asitau Kuru (right). Note the crushed ridges at either end of the cores due to the concurrent impact of hammer and anvil. The arrows denote the largest flake scars on each core. Scale is 1 cm.

**FIGURE 6:**
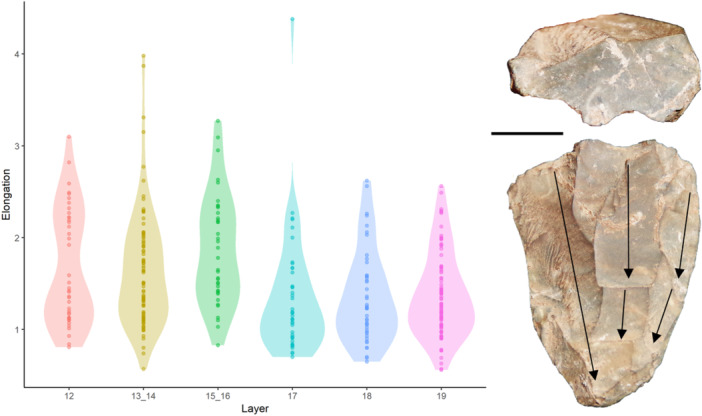
Left: violin plot of flake elongation for Panga ya Saidi; Right: chert laminar bladelet core from layer 13 (late MIS4) at Panga ya Saidi. Note the bimodal blade distribution for layers 12–16 and the unimodal distribution for layers 17–19. Note the elongate parallel scars on the core (shown with black arrows) such that the ridges left by one blade removal guide further blade removals. Adapted from [17]. Scale is 1 cm.

Bipolar knapping is a common, but not universal, feature of miniaturization at Panga ya Saidi and elsewhere in the African Later Stone Age because of its effectiveness in knapping the kinds of small clasts that fine‐grained materials typically occur in, or are reduced to (Figure 5) [37]. Likewise, laminar bladelet production (Figure 6) may be a common, but not universal feature of miniaturization, because it produces long cutting edges per mass of stone at small sizes [38, 39] and because smaller blades have greater retooling tolerance in compound tools [40]. Importantly, at Panga ya Saidi, laminar bladelet production is a characteristic of the early miniaturized assemblages in MIS4 [17] (Figure 6). The MIS4 occupation also features the world's oldest known puka shell bead [22], a type made from water‐worn *Conus* shell spires.

### 2.2 Southern and Western Africa

South and west of Panga ya Saidi, miniaturized Later Stone Age (LSA) assemblages appear slowly, perhaps spreading by a process of diffusion. At Border Cave, near the northeast coast of South Africa (Figure 3), the LSA begins ~43 ka with a similar shift as at Panga ya Saidi from predominantly coarser‐grained rhyolite lithics, to miniaturized pieces on cryptocrystalline quartz and chalcedony produced through bipolar knapping [41]. Farther south on the eastern side of southern Africa, the MSA‐LSA transition is characterized by an increase in quartz at the expense of hornfels in the introduced lithic materials [42, 43]. Assemblages classed as “late MSA” at Sibebe and Holley Shelter dating between 30 and 27 ka in eastern southern Africa are dominated by lithics < 2 cm and bipolar flaking [44, 45]. The early LSA across southern Africa is most commonly associated with bipolar flaking and appears to follow a north‐east to southwest cline of spread; appearing first at Border Cave and latest on southwestern coast [46]. The late survival of the MSA into the Holocene in west Africa [47] might also be explained by a slow diffusion of the LSA from the east coast. Lithic assemblages dominated by the bipolar flaking of small quartz flakes appear at Mumba in the interior of east Africa 57 ka [48] and only reach Shum Laka in Cameroon 32 ka (Figure 3) [49]. However, to the north and east of Panga ya Saidi, the spread of miniaturization appears much faster, perhaps reflecting dispersal rather than diffusion (Box 2).

BOX 2 **Taramsa 1.**

While the Nile Valley is often thought to have been the route via which *Homo sapiens* left Africa, no clear dispersal signature has yet been found in the lithics. A potential case is to be found at Taramsa Hill 1 in southern Egypt (Figure 3). The site is a flint quarry that was used repeatedly across MIS7‐3. Extraordinary refit sequences allowed the reconstruction of an unusual bidirectional blade technology on bifacial cores in layers dated to MIS4 [50, 51]. This is succeeded by a more typical Levallois blade technology in early MIS3. Due to the large volume of excavated material, lithics < 15 mm were not systematically recorded. However, the material from early MIS3 is still distinctive in its small size: “Most striking is the reduced average length in comparison to the other assemblages from Taramsa 1” ([51]: 228). Pieces < 15 mm are described as “abundant” ([50]: 123) and “very numerous” ([50]: 135) in early MIS3, so the assemblage overall may be miniaturized.

## 3 The Middle East

### 3.1 Shi'bat Dhiya 1

In Arabia, there are very few sites from MIS4 and early MIS3. One exception is Shi'bat Dhiya 1 in Yemen (Figure 3), where a dense layer of refitting lithics was discovered dating to 55 ka (Table 1) [52]. These artifacts are commonly ascribed to the Middle Paleolithic, as they feature the Levallois technique. However, many lithics were knapped using a laminar system, entailing continuous large removals from the main flaking surface with the ridges from previous blades guiding the next blade. The resulting assemblage features extremely elongate parallel‐sided blades, beyond those seen in other Middle Paleolithic assemblages. The rhyolite used to knap 94% of the lithics in the assemblage is described as abundant, easily available, and a particularly high‐quality, fine‐grained variety. Refitting clasts are well over 10 cm in maximum dimension. Given this size, availability, and abundance, it is noteworthy that 82% of the assemblage is smaller than 20 mm [60]. The rhyolite is described as tough, while the refits indicate rapid burial, so the small debitage is unlikely to have been caused by trampling. The dominance of this small flake component, coupled with the fine‐grained nature of the rhyolite, means the assemblage could certainly be described as miniaturized. Southern Arabia may have acted as a refugium for hominin populations in the peninsula during the aridity of MIS4 [60], with one genetic study suggesting non‐African *Homo sapiens* underwent a prolonged period of isolation and adaptation in Arabia at this time [7]. Surface sites of miniaturized Levallois from Dhofar in southern Arabia have also been suggested to reflect a southern dispersal out of Africa [61].

**TABLE 1:** Key age estimates in ka related to early miniaturized lithics in southern Asia, Wallacea, and Australia.

Site	Cryptocrystalline Miniaturization	Stratigraphy	Type	Lab code	+1σ	−1σ	Median	Ref
Shi'Bat Dhyia 1	N/A	Below occupation	OSL	SD1‐08/OSL11	61	51	56	[52]
Shi'Bat Dhyia 1	Small flakes	Above occupation	OSL	SD1‐08/OSL19	60	50	55	[52]
Shi'Bat Dhyia 1	Small flakes	Above occupation	OSL	SD1‐08/OSL10	59	49	54	[52]
Dhaba	Bladelets	3 J—early microblade	OSL	Dhab3 OSL2	51.3	45.9	48.6	[53]
Dhaba	N/A	2E—below early microblade	OSL	Dhab2 OSL2	49.5	45.5	47.5	[53]
Mehtakheri	Bladelets, small flakes	1.23—microblade bearing	OSL	MHK‐07‐05	51.9	42.1	47	[54]
Mehtakheri	N/A	1.25—below microblades	OSL	MHK‐09‐10	47.7	40.9	44.3	[54]
Mehtakheri	N/A	2.3—underlying microblades in layer	OSL	MHK‐07‐03	61.3	49.7	55.5	[54]
Fa‐Hien Lena	Small flakes, bipolar reduction, & backed microliths	Early occupation, context 34	C14	Beta‐354918	48.046	45.028	46.537	[55]
Kitulgala Beli Lena	Small flakes, bipolar reduction, & backed microliths	Early occupation, context 23	C14	OxA‐37483	44.902	42.593	43.7475	[23]
Makpan	Small flakes, bipolar reduction	Early occupation, layer 18	C14	SANU‐53609	44.533	37.823	41.178	[56]
Laili	Small flakes	Initial occupation, layer 20	C14	WK‐51314	44.340	43.305	43.853	[18]
Laili	Small flakes	Early occupation, layer 19	OSL	L19‐5	52.301	47.299	49.8	[18]
Laili	N/A	Underlying initial occupation, top of layer 21	OSL	L19‐4	56.301	46.899	51.6	[18]
Asitau Kuru	Small flakes, bipolar reduction	Early occupation, layer 8	C14	SANU‐56310	45.348	42.488	43.918	[21]
Widgingarri 1	Small flakes, bipolar reduction	Early occupation, phase 1	OSL	WIDG‐8	51.8	47.2	49.5	[24]
Carpenters Gap 1	Small flakes, bipolar reduction	Initial occupation, layer 8	C14	ANUA‐7616	45.604	42.894	44.249	[57]
Carpenters Gap 1	Small flakes, bipolar reduction	Initial occupation, layer 8	C14	ANUA‐7617	45.365	42.713	44.039	[57]
Karnatukul	Small flakes	Early occupation, base of layer 5	C14	WK‐44287	45.3	41.79	43.545	[58]
Warratyi	Small flakes	Early occupation, unit 4	OSL	ERS‐7	45.2	40.4	42.8	[59]
Warratyi	Small flakes	Early occupation, unit 4	C14	OZQ616	49.213	46.301	47.757	[59]
Warratyi	Small flakes	Early occupation, unit 4	C14	Wk‐39526	49.12	45.822	47.471	[59]

*Note:* C14 dates are given calibrated as reported in the references.

### 3.2 The Levant

Systematic production of backed microliths was a feature of knapping throughout the Upper Paleolithic and Epipalaeolithic of the Levant, though such tools do not always dominate assemblages [62]. The Initial Upper Paleolithic is first manifested in the Levant by the Emiran industry, characterized by a combination of Levallois and laminar blade knapping on chert to produce Levallois, bladelet, and Emireh points (the latter having bifacially thinned butts) [63]. One of the earliest occurrences of the Emiran is at Boker Tachtit in the southern Levant where radiocarbon dates suggest an age of ~50 ka [64]. This age is at the limit of radiocarbon dating so may be an underestimate, with optically stimulated luminescence (OSL) ages from the same layers suggesting occupation from the beginning of MIS3, 57 ka. Either way, there is considerable overlap in age with nearby Middle Paleolithic sites in the southern Levant such as Tor Faraj and Farah II [64]. Notably, flint is abundant around Boker Tachtit but 73% of Emiran AH‐B assemblage is < 20 mm [64]. A *Homo sapiens* cranium from the Levant has been dated to 55 ka at Manot Cave and critically it has the parietal bossing that distinguishes later *Homo sapiens* from earlier out‐of‐Africa specimens [65]. Fossils from multiple sites attest to Neanderthal occupation in the Levant during MIS4‐3 [66], so this is a likely location for the interbreeding episode between the two species evident in all living non‐Africans. Admixture dating of recently introgressed Neanderthal aDNA from IUP *Homo sapiens* specimens in Europe, indicate this interbreeding must have occurred in the window 44–51 ka [67, 68].

## 4 Southern Asia

### 4.1 The Indian Sub‐Continent

Mainland India shows a distinct change in its Late Pleistocene lithic sequences from 48 ka. At the site of Dhaba in central India (Figure 3) there is a shift from Levallois technology on limestone, mudstone, and chert, to laminar bladelet technology on quartz, chalcedony, chert, and agate ~48 ka (Table 1) [53]. Laminar bladelet technology on cryptocrystalline materials is found elsewhere in India from this time such as the chalcedony artifacts at Mehtakheri dated from 47 ka (Figure 3 and Table 1) [54]. Combining complete flakes and blades from this site shows a mean artifact length of < 20 mm.

In Sri Lanka there is no evidence of laminar bladelet technology. However, radiocarbon dating shows the caves of Fa‐Hien Lena and Kitulgala Beli Lena (Figure 3) were first occupied 47–44 ka (Table 1) by people making backed microliths, with their material culture also featuring puka shell beads [23, 69]. Lithic technology at these sites is characterized by the bipolar reduction of quartz pebbles to produce miniaturized flakes (Figure 1) [23, 55]. The unifying element of Paleolithic material culture in the Indian Sub‐Continent over the last 47 ka is thus neither laminar bladelet nor bipolar reduction, but rather lithic miniaturization.

### 4.2 Southeast Asia

Until recently, there was no clear lithic technological signal of *Homo sapiens* arrival in mainland Southeast Asia. That situation has now changed with the identification of miniaturized lithics produced through bipolar knapping of tektites (meteoric glass) from 43 ka at Yahuai Cave in southern China (Figure 3) [70, 71]. Yahuai Cave thus provides an articulation point between miniaturized assemblages in the Indian Sub‐Continent and Wallacea.

## 5 Wallacea

### 5.1 Sulawesi

Wallacea presents a useful test of human dispersal as it consists of a series of independent islands that were never connected to mainland Southeast Asia or Sahul, even during the low sea‐level stands of the Late Pleistocene (Figure 3). The largest of these islands, Sulawesi in north‐western Wallacea, has two cave sites showing a transition in their stone artifact sequences. At Leang Burung 2 (Figure 3), large limestone lithics dated to > 35 ka, underlie an industry of smaller chert artifacts featuring bipolar cores [72]. Nearby Leang Bulu Bettue presents a similar shift ~40 ka from large coarser‐grained limestone and volcanic artifacts to smaller chert ones including bipolar flaking, concomitant with the extinction of elephant species and the introduction of aquatic mollusc subsistence [73].

### 5.2 Flores and Alor

In southern Wallacea, Liang Bua on Flores island (Figure 3) shows a switch in lithic material preference from silicified tuff to a more siliceous and finer‐grained chert [74] between 50 and 47 ka, across layers separated by a volcanic eruption [75]. This switch coincides with the change from *Homo floresiensis* to *Homo sapiens* and the extinction of pygmy elephant and giant stork. On neighboring Alor island, miniaturized lithics in the form of fine basalt, chert, and obsidian, reduced using bipolar and core‐on‐flake methods are evident from 40 ka at Makpan Cave (Table 1) (Figure 3) [76]. These lithics occur alongside maritime subsistence, particularly sea urchins in the early occupation [56].

### 5.3 Timor

Timor is the last major island in south‐eastern Wallacea before Sahul and would have required crossing the strong currents of the Indonesian throughflow to reach it from the west [77]. Unlike, Flores and Sulawesi there is no Lower Paleolithic known from Timor, but several sites show early human occupation characterized by miniaturized lithics from 44 ka (Figure 1 and Table 1). Unusually for Wallacea, the Laili rockshelter (Figure 3) has evidence for absence of human occupation in sediment dated from 62 to 52 ka [18]. When occupation with miniaturized lithics begins in the overlying layer, it is dense and features a much higher proportion of aquatic fauna compared with the layers above, suggesting an *en masse* maritime dispersal. While the radiocarbon date for this initial occupation is 44 ka, an OSL date for the layer above of 50 ka suggests the possibility of an older chronology (Table 1). Miniaturized lithics alongside aquatic fauna including tuna are also evident in the early occupation of Asitau Kuru on the eastern tip of Timor (Figures 3 and 5) [21, 78]. Stable isotopes on *Homo sapiens* teeth indicate a particular reliance on a marine diet in this early occupation [79], with puka shell beads also collected unlike in the later occupation at the site [80].

**FIGURE 7:**
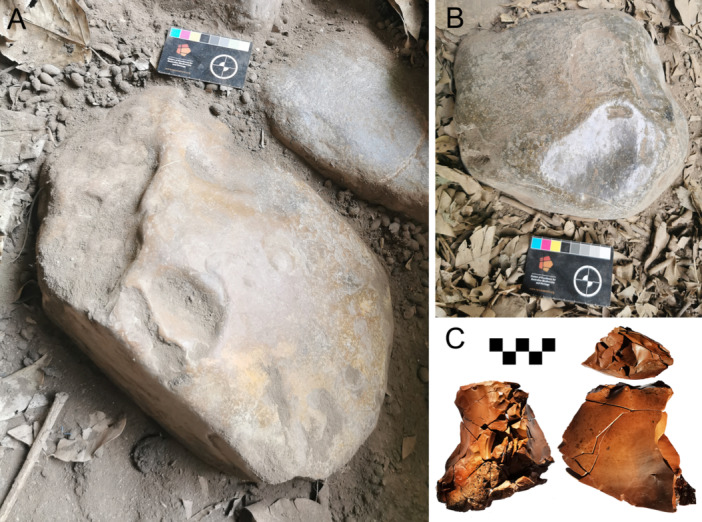
(A and B) Unknapped chert boulders on the current surface at Laili (scale 8 cm); (C) A 70‐piece refit sequence from the initial occupation of layer 20 (scale 5 cm).

**FIGURE 8:**
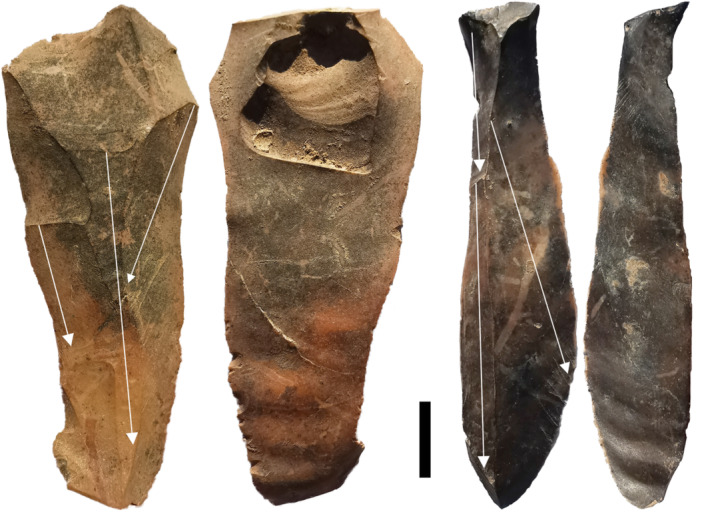
Laminar blades from the MIS3 occupation at Laili. Note the previous scars running along the length of the blades (shown by arrows) creating ridges which have guided the removal of the blades themselves. Scale is 1 cm.

The lithics from Laili and Asitau Kuru are instructive for understanding miniaturization. At Laili miniaturized lithics occur despite the abundance of large cherts clasts (Figure 7) in a river < 500 m from the site [18]. Indeed, a refit sequence shows a large chert flake was reduced into at least 70 further pieces (Figure 7), with three third generation core‐on‐flakes (core‐on‐flakes struck from core‐on‐flakes, struck from a core‐on‐flake) indicating that numerous small flakes rather than a single large one was the goal of knapping. Use‐wear analysis shows that miniaturized pieces were indeed used for cutting tasks [18]. Bipolar reduction was common throughout the Laili assemblage, while the production of occasional laminar blades may have been afforded by the large cryptocrystalline clasts (Figure 8), though this does not appear to have been a major component of the technology. The length of the largest complete flake scar on cores gives an indication of the size of the flakes that were being produced at the point where the core was no longer considered productive and discarded. Analysis of this data at Asitau Kuru, featuring multifacial, discoidal, bipolar, and core‐on‐flake pieces, shows that nearly all had largest flake scars < 20 mm long (Figure 5) [21] so miniaturized lithics were a desired end‐product.

## 6 Sahul

### 6.1 Madjedbebe

The rockshelter of Madjedbebe at the foot of the Arnhem Land plateau in northern Australia (Figure 3) contains the earliest evidence of human occupation in the continent of Sahul, with OSL dates of 65 ka [81]. The lithics from this early phase are characterized by bifacial thinning flakes and the use of microcrystalline quartzite and silcrete. Groundstone axes in this early phase suggest the early occupants of the shelter were *Homo sapiens* as this technology has never been associated with another species. The only other site with a pre‐50 ka occupation age thus far known in Sahul is Nauwalabila I, also in Arnhem Land [82]. Overlying the early occupation at Madjedbebe is a layer dated from 49 ka with the lithics characterized by bipolar flaking of quartz [81].

### 6.2 Australia

From 49 ka, sites appear across Australia. In the Kimberley, early occupations at Minjiwarra and Widgingarri 1 have OSL dates of ~49 ka [24, 83], while initial occupations at Carpenters Gap 1 and Riwi have radiocarbon dates of ~45 ka (Figure 3 and Table 1) [57, 84]. Lithics from Widgingarri 1, featuring unifacial, multifacial, and bipolar reduction, are miniaturized throughout this sequence [24] (Figure 1). Likewise, lithics from Carpenters Gap 1 are characterized by bipolar flaking of small pieces of crystal quartz [57].

At Karnatukul in the Western Desert an assemblage dominated by cryptocrystalline lithics in the 6‐2 mm maximum dimension range is dated by radiocarbon from 45 ka (Table 1) [58]. Initial occupation at Warratyi rockshelter in south‐central Australia features an assemblage dominated by quartz lithics with average weight < 2 g, and radiocarbon dates of 47 ka (Table 1) [59].

### 6.3 New Guinea

Similarly to Australia, evidence for human presence in New Guinea is widespread from 46 ka: Radiocarbon dates indicate the occupation of the Ivane Valley in the Highlands from this time and Buang Merabak in the Bismarck Archipelago from 44 ka [85, 86]. Estimates from modern genomic diversity for the colonization of Sahul within the last 50 ka [4, 87] accord with archeological evidence for widespread occupation of the continent. However, these genetic estimates for the colonization of Sahul do not fit with the early dates for Madjedbebe [88]. One analysis suggests that an early otherwise extinct Out‐of‐Africa population makes a 4% contribution to the genomes of modern Papuans [89], with such a population perhaps represented by the early occupation of Madjedbebe. Mitochondrial DNA evidence using a slower mutation rate estimate, also suggests two different founder populations for Sahul and a pre‐60 ka age for the modern diversity [90]. Gene flow into Altai Neanderthals from a *Homo sapiens* population distinct from extant non‐Africans suggests an early eastward dispersal of our species [91]. This introgression occurred after the split of Altai Neanderthals from European Neanderthals ~110 ka, suggesting the dispersal took place in late MIS5.

## 7 Southern Europe

### 7.1 Grotte Mandrin

Grotte Mandrin in southern France (Figure 3) presents the unusual situation of an IUP assemblage sandwiched in between Middle Paleolithic layers [92]. OSL dating puts this occupation at 54 ka, while teeth indicate the presence of our species. Both the IUP layer and the surrounding Middle Paleolithic are dominated by small lithics < 2 cm, however the latter may due to the preservation of retouching flakes from the creation of larger tools such Quina scrapers in the layer below and ventrally retouched points in the layer above [92]. In the IUP layer, use‐wear analysis has shown that small points were themselves the desired end‐products as arrowheads [93]. A parallel has been drawn between the production of these small points and associated blade points with those in the earliest IUP assemblage from Ksar Akil in the Levant [94].

### 7.2 The Uluzzian and Chatelperronian

The Uluzzian and Chatelperronian are distinctive stone tool industries post‐dating the Middle Paleolithic in Italy and France/northern Iberia, respectively. Their ages span 45–40 ka, and both are somewhat controversially associated with *Homo sapiens* [95, 96, 97]. Uluzzian stone artifacts are characterized by the bipolar flaking of small flint pebbles to produce small flakes and bladelets, which were sometimes retouched into backed crescents and used as arrow tips [98, 99]. Such features are resonant with many assemblages of the African LSA (e.g., [17, 46]). Chatelperronian knappers were in the unusual situation of having large clasts of cryptocrystalline flint available (similar to Laili but over a much larger area). Blades and bladelets were produced by laminar reduction and often used without retouch, with the most distinctive retouched type being backed points on blades [96].

### 7.3 The Aurignacian

The Aurignacian industry is unambiguously associated with our species across much of Europe and dates from ~42 ka [98, 100]. While the Aurignacian overall is not miniaturized, its most characteristic lithics, particularly in the initial Proto‐Aurignacian manifestation in southern Europe are Dufour bladelets (ventrally retouched) [101] and carinated scrapers [98], the latter now understood to be a type of bladelet core‐on‐flake [102].

## 8 Why Miniaturization?

### 8.1 A Miniaturized Dispersal?

The above review indicates the rapid appearance of miniaturized assemblages across southern Asia and over to Sahul between 60 and 40 ka. Some elements of miniaturization, such as small retouched tools, are also evident in the Upper Paleolithic of the Levant and southern Europe [61, 101, 103]. The transition is so rapid that miniaturized assemblages appear in Australia at approximately the same time as southern Asia, suggesting this may be an adaptation to a climatic change rather than a dispersal signature. However, there is no candidate climatic change that would have had a similar influence on the diverse environments occupied by humans with miniaturized assemblages at this time. For example, wetter global climatic conditions in the early MIS3 interstadial may be characterized as amelioration in mid‐latitude Arabia and South Asia, but in equatorial Southeast Asia they represent dense forests and reduced landmass [104]. The persistence of miniaturization over long sequences with fluctuating environmental proxies [17, 24], does not accord with an ecological explanation. Another possibility is that miniaturization occurred as the result of population increase placing pressure on stone resources in procurement territories [105, 106]. However, at Panga ya Saidi, the switch to miniaturization occurs at the nadir of occupation intensity in the sequence [17], while on Alor and Timor miniaturization occurs at the outset of occupation sequences [76], indicating it cannot be a local response to population growth.

The earliest miniaturized assemblages are found in Africa at Panga ya Saidi and then outside of Africa at Shi'bat Dhiya 1 in adjacent Yemen, giving them a temporal west‐east cline from east Africa to southern Asia in accordance with an out‐of‐Africa dispersal event. More sporadically, puka shell beads are documented on this west‐east trajectory, first at Panga ya Saidi in east Africa, and later at Fa Hien Lena in Sri Lanka and Asitau Kuru in Timor‐Leste. The timing of miniaturization in Eurasia, Wallacea, and Sahul accords with genetic estimates for *H. sapiens* dispersal 55–45 ka [4, 5, 6, 7, 67, 87]. Concomitantly, the timing of miniaturization in coastal east Africa accords with the genetic estimates for an out‐of‐Africa migration event 75–55 ka [4, 5, 6]. A second wave of dispersal to Sahul could explain genetic evidence for a small contribution of an early dispersal and the change in materials and technology 49 ka at Madjedbebe. If miniaturization is a dispersal signature, then it requires explanation as to why it should have been so rapid and dominant.

### 8.2 Miniaturized Function

The key element of miniaturized lithics is perhaps not so much their size but the focus on cryptocrystalline rocks when larger package knapping materials are available. Experimental evidence shows a trade‐off between durability and sharpness in lithic materials, with cryptocrystalline rocks like quartz being sharper, and coarser‐grained rocks like basalt being more durable [20]. Experiments also show that the sharpness of stone flakes drops off rapidly upon initial use [107]. Thus, if you want the sharpest edges, it is better to create many small flakes from a clast, then use them briefly and replace them. One of the advantages of miniaturization is the greater length of cutting edge produced for a given mass of stone [39, 108]. A further feature of miniaturized assemblages suggests the priority of sharpness over stone tool longevity. Stone flakes are sharpest without retouch as the simpler topography (and typically lower angle) of the unretouched edge creates less friction with the substrate material. Rates of retouch are often low in miniaturized assemblages in general, with the most common form of retouch being backing on the hafted edge rather than shaping/resharpening of the cutting edge (e.g., [17, 46, 96]). A prioritization of sharpness leads to the question of which functions require the sharpest lithics?

One probable function for both backed microliths and small points in the Paleolithic is as arrow tips [93, 109]. These small sharp tools have the advantage of enhancing the penetration of arrows without excessively weighing them down [19]. However, lithic miniaturization and backing is evident in recent times in areas such as Australia where bow‐and‐arrow technology was not ethnographically documented [24]. Backed microliths were also likely used as barbs on spear shafts where they enhance the damage of the weapon without overweighting it [110]. But a few backed barbs and arrowheads cannot account for the thousands of unretouched lithics in miniaturized assemblages. Furthermore there are many miniaturized assemblages that lack backed artifacts altogether, including the MIS4 and late MIS3 occupation at Panga ya Saidi [17] and sites in eastern Wallacea [34]. A function as inserts in compound tools may apply to miniaturized lithics more generally, with ethnographic examples of multiple quartz flakes hafted in series on Australian tools including Death Spears and Taap Knives [16]. Such compound tools may explain a proportion of miniaturized assemblages but are unlikely to account for all of them, with ethnographic evidence also pointing to a further function of miniaturized lithics that uses dozens at a time: body modification. Hair‐cutting and scarification are two widely attested functions for miniaturized lithics across diverse ethnographic settings including east Africa, the Andaman Islands, New Guinea, and Australia [16, 111]. Cutting hair requires the sharpest edges due to its pliable nature; while making a clean cut for neat scarification designs also involves the sharpest edges. When miniaturized lithics are used in this way they are typically employed for one or two cuts then discarded and replaced [16].

### 8.3 Miniaturized Advantages?

The bow‐and‐arrow would have given its makers a formidable advantage over those without this technology and may be one reason for the rapid success of the dispersal of our species from 55 ka [19, 93]. Likewise, compound tools represent a significant technological threshold that could have enhanced various food getting tasks such as fishing with harpoons or cereal gathering with sickles.

A social explanation for dispersal success may lie in the body modification function of miniaturized lithics. In traditional societies, hairstyles and scarification designs typically denote a particular status to the wearer, outwardly signifying their adherence to group norms to people beyond the residential group [112]. Body ornamentation in the form of paint is a moderately costly signal of identity that is attested from the late Middle Pleistocene [113]. Hairstyles, and in turn scarification, represent significantly higher costs of identity signal due to their increasing permanence, and, in the case of scarification, infection risk; therefore these are more robust indicators of commitment. Miniaturization may represent a new threshold in identity signaling which non‐residential group members could use in identifying unknown members of a broader tribal supergroup as reliable allies. The existence of such supergroups is suggested by genetic diversity comparisons showing Neanderthals had much smaller group sizes than Upper Paleolithic *Homo sapiens* [114]. The repeated movement of obsidian lithics over distances > 500 km shows large social scales were a feature of Upper Paleolithic societies (e.g., [115]). Supergroups may in part explain the dispersal success of our species: Reducing competition between neighbors; transmitting adaptive information over long distances; and having the option to retreat to friendly country when moving into new territory. A parallel may be drawn with unicolonial ant societies, who form supercolonies with non‐aggression between different daughter nests based on broader group recognition [116]. Such ants are ecologically dominant when introduced as invasive species, with five of 17 of the world's most invasive invertebrates being unicolonial ants.

## 9 Conclusion

Lithic miniaturization provides a repeated signature of *Homo sapiens* behavior from ~50 ka in Sahul [24], through Wallacea [76], and Southeast Asia [70]. It is a feature of both Sri Lankan [55] and Indian assemblages [54], while laminar blade technology is only represented in the latter. Miniaturization is distinct from the lithic technology of earlier hominins in these regions, including the Middle Paleolithic Levallois of earlier *Homo sapiens* [53]. Both miniaturization and laminar blade technology are manifested at Shi'bat Dhiya 1 in Yemen [60] and Boker Tachtit in the southern Levant [64]. MIS3 *Homo sapiens* sites to the north (Upper Paleolithic) are characterized by laminar blade technology but do not tend to be miniaturized, although they do typically feature retouched microliths. The south‐western margins of Asia thus represent the coalescence of laminar blade technology and miniaturization, as well as their earliest manifestation outside of Africa. Importantly, both these features can be traced back to MIS4 in eastern Africa at Panga ya Saidi [17], giving a window of around 13,000 years in which an out‐of‐Africa dispersal could have occurred.

To square genetic evidence for most of extant non‐African ancestry diverging rapidly in MIS3 [67, 68, 87] with fossil and archeological evidence for our species in Eurasia in MIS5, requires a compelling explanation for the competitive advantage of later *Homo sapiens*. The implications of lithic miniaturization in bows‐and‐arrows, compound tools, hair‐styling, and scarification, may provide such a reason in combined utilitarian and social dominance. A common underlying cognitive process of abstraction has been suggested for these behaviors [16]. Endocranial globularity increases between early *Homo sapiens* outside of Africa and later representatives of our species [117], with the earliest manifestation of the modern form being the 55 ka specimen from Manot Cave in the Levant [65]. The change in neural architecture associated with this increase in globularity may provide a mechanistic explanation for the emergence of behaviors reflected in lithic miniaturization.

## Supporting information

Miniaturization comparison 2.

## Data Availability

The data that support the findings of this study are available in the supporting material of this article.
